# Supplementary material for: Nutritional Quality of Food and Beverages Offered in Supermarkets of Lima According to the Peruvian Law of Healthy Eating
Source: Nutrients. 2020 May 22;12(5):1508. doi: 10.3390/nu12051508 (PMC7285082; doi:10.3390/nu12051508)
Supplement: Supplementary file 1 [file nutrients-12-01508-s001.zip › Supplementary Material 2.docx]

**Table S2.** Categories of food and beverages adapted from Chilean studies to the Peruvian context.

|  | **Categories from Chile*** | **Study Categories** | **Examples** |
| --- | --- | --- | --- |
| 1 | Processed meats | Processed meats | Ham, hamburgers, nuggets, canned or breaded meat, fish or seafood |
| 2 | Fish and seafood |  |  |
| 3 | Sweets | Candies | Hard candies, chocolates, chewable candies |
| 4 | Deserts/ice cream | Deserts | Pudding, ready to eat desserts |
|  |  | Ice Cream | Milk or water based ice cream |
| 5 | Dairy sweets | Dairy drinks | Yogurt, chocolate milk, milk shake |
| 6 | Dairy savory |  |  |
| 7 | Beverages | Beverages | Juices, nectars, soda, flavored drinks |
| 8 | Sauces/spreads | Sauces, spreads and dressings | Mayonnaise, ketchup, mustard, salad dressing, tomato sauce, soy sauce |
| 9 | Savory bread and bakery | Bakery products | Packaged breads, cookies, pastry, packaged cakes |
| 10 | Sweet bread and bakery |  |  |
| 11 | Sweets sauces spreads | Sweet spreads | Jam, hazelnut, peanut butter |
| 12 | Breakfast cereals/bars | Cereals | Cereals, granola, oat, muesli, granola bars |
| 13 | Savory snacks | Snacks | Potato chips, cheese snacks, puffed snacks, nuts and seeds |
| 14 | Sweet snacks |  |  |
| 15 | Processed food |  |  |
| 16 | Ready-to-eat-foods | Ready-to-eat-meals | Instant soups, instant or ready to eat foods, pasta dishes, pizza. |
| 17 | Processed vegetables |  |  |
| 18 |  | Condiments and spices | Dried pepper flakes, dried peppers, spice mixed, marinades for turkey, pork, meats. |

* *Kanter R, Reyes M, Vandevijvere S, Swinburn B, Corvalan C. Anticipatory effects of the implementation of the Chilean law of food labeling and advertising on food and beverage product reformulation. Obes Rev. 2019; 1-12.*
