# Supplementary figures and images for: Nutritional Quality of Food and Beverages Offered in Supermarkets of Lima According to the Peruvian Law of Healthy Eating
Source: Nutrients. 2020 May 22;12(5):1508. doi: 10.3390/nu12051508 (PMC7285082; doi:10.3390/nu12051508)

**S1**. Example of a photograph group.


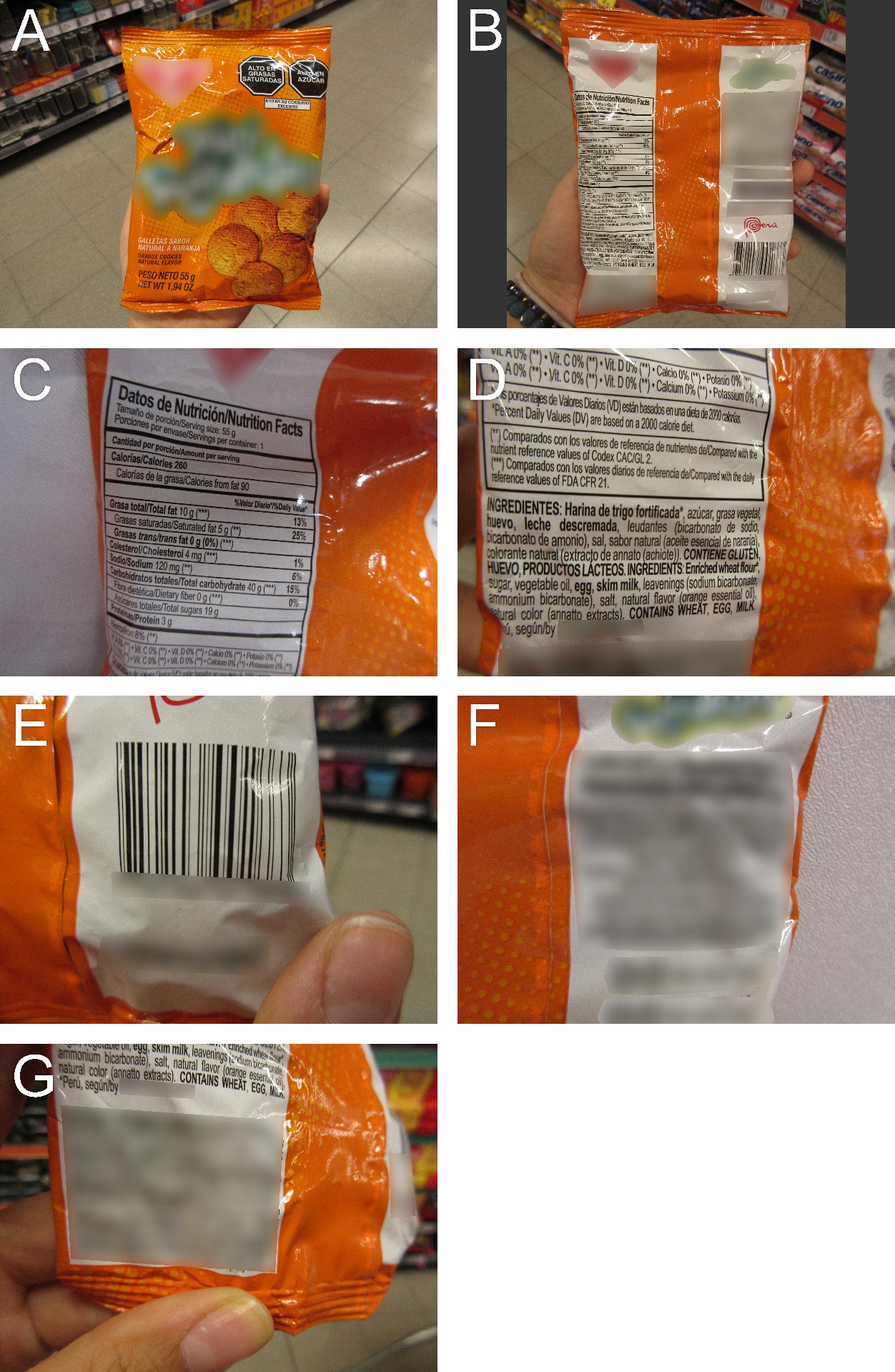

Supplement: Supplementary file 1 [file nutrients-12-01508-s001.zip › Supplementary Material 1.docx]
